# 86. Frequency of Viral Monitoring to Detect Cytomegalovirus Infection and Prevent Disease in Solid Organ Transplant Recipients: An International Multicenter Study

**DOI:** 10.1093/ofid/ofaf695.032

**Published:** 2026-01-11

**Authors:** Anja Lindis Dahl, Oriol Manuel, Kristian Schoenning, Helene Høgsbro Thygesen, Michael Perch, Søren Scwartz Sørensen, Nicolai Aagaard Schultz, Kasper Rossing, Jens Lundgren, Nicolas Johannes Müller, Marie Helleberg

**Affiliations:** Rigshospitalet, Copenhagen, Hovedstaden, Denmark; Lausanne University Hospital and University of Lausanne, Lausanne, Luzern, Switzerland; Rigshospitalet, Copenhagen, Hovedstaden, Denmark; Rigshospitalet, Copenhagen, Hovedstaden, Denmark; University of Copenhagen, Copenhagen, Hovedstaden, Denmark; Rigshospitalet, Copenhagen, Hovedstaden, Denmark; Rigshospitalet, Copenhagen, Hovedstaden, Denmark; Rigshospitalet, Copenhagen, Hovedstaden, Denmark; Rigshospitalet, University of Copenhagen, Copenhagen, Hovedstaden, Denmark; Zåurich University Hospital, Zürich, Zurich, Switzerland; Rigshospitalet, Copenhagen University Hospital, Copenhagen, Hovedstaden, Denmark

## Abstract

**Background:**

Cytomegalovirus (CMV) is a major cause of morbidity among solid organ transplant recipients (SOTr). The risk of CMV disease during the first post-transplant year can be reduced by antiviral prophylaxis or pre-emptive therapy (PET), i.e. monitoring for early CMV replication in patients not receiving prophylaxis. For patients completing prophylaxis, surveillance after prophylaxis (SAP), may be used. However, the optimal frequency of CMV monitoring for PET/SAP remains unclear, and it is debated whether SAP is needed at all.

**Table 1:**
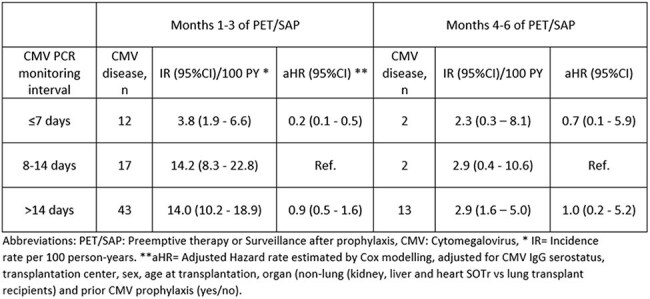
Interval between CMV PCR monitoring during PET/SAP follow-up and risk of CMV disease at time of diagnosis of CMV infection.

**Table 2:**
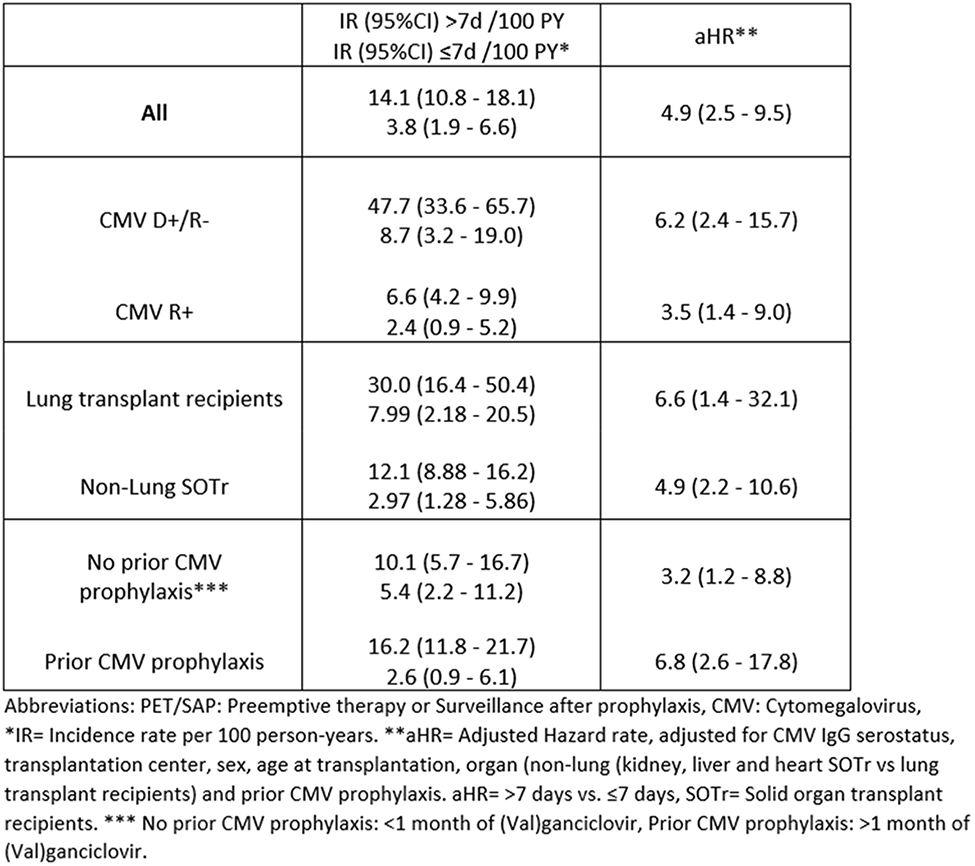
Incidence rates and adjusted hazard rates of CMV disease associated with CMV PCR monitoring of > 7 vs. ≤ 7 days for prevention of CMV disease during the first 3 months of PET/SAP.

**Methods:**

All adults who received a SOT in Zürich/Lausanne (2010-2022) or Copenhagen (2012-2021), CMV IgG donor (D) and/or recipient (R) positive were included and followed for 6 months after stop of CMV prophylaxis or 6 months after transplantation if no prophylaxis was given. Cumulative incidences of CMV disease at time of diagnosis of CMV infection (i.e. failure of applying a PET/SAP strategy) were examined in analyses including death and re-transplantation as competing risks. Multivariable Cox regression investigated associations between CMV PCR monitoring intervals and risk of CMV disease. Cox model-derived number needed to test (NNT) to prevent CMV disease with monitoring intervals of ≤ 7 vs > 7 days were calculated.

**Table 3:**
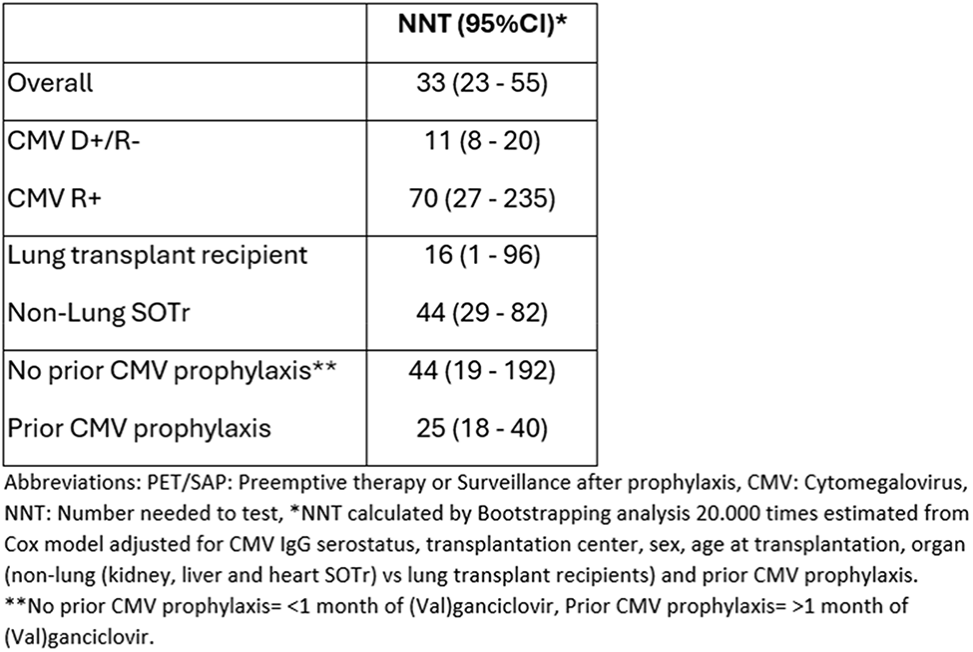
Number Needed to Test for interval between CMV PCR monitoring of ≤ 7 vs. > 7 days for prevention of CMV disease during the first 3 months of PET/SAP.

**Results:**

Of 3,424 SOTr, median age 54 years (interquartile range 43-62), 72 (2.1%) were diagnosed with CMV disease concurrently with CMV infection within the first 3 months of PET/SAP, whereas only 17 (0.5%) cases were seen in month 4-6 (Table 1). A CMV monitoring interval of ≤ 7 vs. 7-14 days within the first 3 months of PET/SAP was associated with significantly lower risk of CMV disease, while the risk did not differ between > 14 vs. 7-14 days (Table1). The increased risk of CMV disease associated with CMV monitoring intervals > 7 days was consistent across all subgroups of SOTr (Table 2). The NNT to prevent one event of CMV disease was 11 (95%-CI: 8-20) for the D+/R- vs. 70 (95%-CI: 27-235) for the R+ SOTr but did not differ according to prior use of prophylaxis (Table 3).

**Conclusion:**

Our finding of significant lower risk of CMV disease associated with frequent CMV monitoring for PET/SAP, supports close monitoring and a hybrid CMV prevention strategy with SAP. Frequent monitoring is more efficient in D+/R- SOTr, as reflected by the lower NNTs for this group.

**Disclosures:**

All Authors: No reported disclosures

